# Evaluation of *Lactobacillus coryniformis* CECT5711 strain as a coadjuvant in a vaccination process: a randomised clinical trial in healthy adults

**DOI:** 10.1186/s12986-016-0154-2

**Published:** 2017-01-05

**Authors:** Noemí Redondo, Esther Nova, Alina Gheorghe, Ligia Esperanza Díaz, Aurora Hernández, Ascensión Marcos

**Affiliations:** Immunonutrition Group (Metabolism and Nutrition Department) – Institute of Food Science, Technology and Nutrition, Spanish National Research Council (ICTAN-CSIC), José Antonio Novais St. 10, 28040 Madrid, Spain

**Keywords:** *Lactobacillus coryniformis* CECT5711, Probiotics, Immune response, Vaccine, Healthy Adults

## Abstract

**Background:**

Although the effects of probiotics on the immune system have been extensively evaluated under disease states, their role in healthy situations remains unclear, since changes are hardly expected under immunological homeostasis. EFSA indicates that vaccination protocols could be used to evaluate the potential role of probiotics to improve the immune response against antigen challenges. The aim of the study was to evaluate the effect of *Lactobacillus coryniformis* CECT5711 (Lc) on the specific immunity of healthy volunteers undergoing vaccination with Hepatitis A virus (HAV).

**Methods:**

One hundred twenty-three healthy adults were randomised into three groups to follow a 6-week (wk) intervention and all received an intramuscular HAV vaccine 2 weeks after starting the intervention: 1) PRO1 received Lc for 2weeks (1 capsule/day; 3 × 10^9^ CFU/capsule) and placebo capsules after vaccination; 2) PRO2 received a daily capsule of Lc (3 × 10^9^ cfu/day) before and after the challenge; 3) Control group (C) received a daily placebo capsule before and after the vaccine. Blood samples were collected at the beginning (visit 1; V1) and after 2 (V2) and 6 weeks (V3) of the intervention. At each visit, lymphocyte subset counts and cytokine levels were analysed. Specific HAV antibodies were analysed at V1 and V3. To evaluate differences between groups, one-way ANOVA with Bonferroni post-hoc test were used regarding lymphocyte subset counts and specific HAV antibodies production, and Friedman test of related samples and Kendall concordance coefficient for cytokines production. Chi square test was used to analyse seroconversion rates.

**Results:**

Specific HAV antibodies were significantly higher in PRO1 (50.54 ± 29.57) compared to C (36.23 ± 16.45) (*P* = 0.017) and showed an intermediate value in PRO2 (41.61 ± 15.74). Seroconversion rates were similar in the three groups (97.3, 92.3 and 97.4% in C, PRO1 and PRO2 respectively). Memory T-helper lymphocytes increased in V3 vs. V1 (*P* = 0.032) in PRO2. No differences were found in cytokine concentrations.

**Conclusion:**

Mixed results have been found regarding the usefulness of Lc supplementation to increase the antigen-specific antibody response to an immune challenge. Clinical trial registration number: EudraCT Number 2016-000183-42. Registered 19 January 2016. Retrospectively registered.

**Electronic supplementary material:**

The online version of this article (doi:10.1186/s12986-016-0154-2) contains supplementary material, which is available to authorized users.

## Background

There is wide evidence about how nutrition affects the immune system and modulates the resistance to infection [1, 2]. Currently, there is a vast research about the role of specific food components in enhancing immune responses against a challenge with the aim to improve health and reduce disease risks [3]. In this line, the interest in probiotics has substantially increased over the last two decades, which are well-defined as ‘live bacteria that offer a health benefit to the host when administered in adequate amounts’ [4]. Probiotics have been shown to exert beneficial effects in health and disease in many studies [5, 6]. In particular, probiotic intake is related to a better control of infectious diseases [7], and in some cases with an improvement of the duration or severity of infections [8, 9]. The mechanism could be related to an interaction between probiotics and intestinal bacteria and thus to the innate and specific host immune cells [10].

The European Food Safety Agency (EFSA) states that vaccination protocols may be allowable in order to evaluate the potential role of probiotic strains on improving the immune response against antigen challenges [11]. In this regard, the stimulation of protective antibody titres could be used under standardized conditions to substantiate a health claim on the function of the immune system related to defence against pathogens [11–13]. In fact, these protocols have been already used in studies with healthy subjects [14, 15]. Lactobacilli are considered potential candidates to develop antigen delivery strategies for immunization [4]; indeed, these bacteria have been included in our diet into many fermented products for centuries. In fact, the main objective of employing lactic acid bacteria as coadjuvants in a vaccination process is to gain a more efficient immune response [9].

The strain used in this study, *L. coryniformis* CECT5711, was isolated from an artisan goat milk cheese [16] and it has been recognised as QPS (*qualified presumption of safety*) by EFSA. This strain has been proven to comply with the main safety criteria [17] and the most important properties for probiotics to exert their effects on the immune system [16, 18]. In addition, it has been related to an improvement of both innate and specific immune response in previous studies in healthy subjects when consumed along with *L.gasseri* CECT5714 [19, 20]. Since vaccine-antibody response is mediated by the activation of both responses, the aim of this study was to find out whether the consumption of this single strain, under a Hepatitis A vaccine model, could induce a vaccine-antibody response and thus be used as a coadjuvant in a vaccination process.

## Methods

### Experimental design

This study is a randomized, double-blinded, placebo-controlled, human intervention trial, which started on May 2012 and finished on April 2013. A 2-weeks run-in was performed prior to the intervention and followed during all the study. During this time subjects were asked to avoid any fermented food, probiotics or prebiotics consumption. All volunteers were vaccinated at week 2 of the study in the medical service of the “Spanish National Research Council (CSIC)”, with a “HAVRIX 1440” inactivated Hepatitis A vaccine. The intervention lasted 6 weeks (wk), which was divided into a pre-vaccination period (2weeks before the intramuscular vaccine), plus a post-vaccination period (4weeks following the vaccine). Although the between-subject variability in response to vaccination is normally quite high, the period between vaccination and the plateau phase of the response starts from about 3 weeks [12]. For this reason, the measurement of antibody production was established after 4 weeks of vaccination. After an overnight fast, blood samples were collected at the start of the intervention or visit 1 (V1), after 2 weeks or visit 2 (V2) and after 6 weeks or visit 3 (V3).

### Subjects

**Fig. 1:**
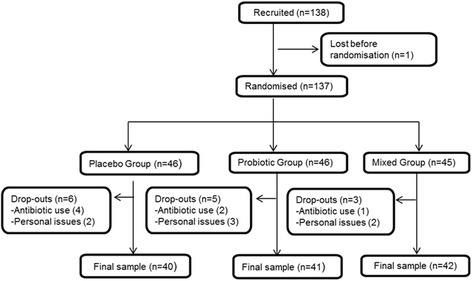
Flowchart of participating subjects

Sample size calculation was performed to demonstrate a 5% difference in specific Hepatitis A antibody titers with a power of 80% and a significance level of 0.05. Under these assumptions, based in previous published work [21], a sample size of at least thirty-six subjects per group would be required. In total, 138 healthy adults started the study, but only 123 finished the trial (Fig. 1). The main dropout reasons were antibiotic treatment or personal issues. The recruitment of the volunteers was carried out through advertisement and on-line services. The exclusion criteria were frequent gastrointestinal, metabolic and immunological disorders (lactose intolerance or food allergies), antibiotic treatment during two months prior to the intervention or pregnancy. All volunteers were young adults (aged 20–45 years), showing a normal body mass index (BMI) (between 18.5 and 24.9 kg/m^2^) [22], who reported not to have been vaccinated or had suffered from Hepatitis A.

Volunteers included in the study were randomly allocated into one of the three groups established: 1) PRO1 received a daily capsule of Lc for 2weeks (3 × 10^9^ colony forming units ([cfu]/capsule) and after vaccination received placebo capsules with maltodextrin; 2) PRO2 received a daily capsule of Lc (3 × 10^9^ cfu/day) before and after the vaccine; 3) Control group (C) received a daily placebo capsule with maltodextrin before and after the vaccine. A stratified randomization procedure was followed using a random number generator with an informatics program and sex, age and BMI as potential covariates. The capsules were kept in the fridge and ingested after dinner. Baseline characteristics of the volunteers are described in Table 1.

**Table 1:** Baseline characteristics of the volunteers in each group in the immune general assessment (A) and in the Hepatitis A-specific antibody analysis (B)

	Control		PRO2		PRO1	
	A (*n* = 40)	B (*n* = 38)	A (*n* = 41)	B (*n* = 38)	A (*n* = 42)	B (*n* = 37)
Men	13	13	12	11	13	10
Women	27	25	29	27	29	27
Age (mean ± SD, years)	26.7 ± 5.8	26.2 ± 5.2	27.1 ± 6.0	26.8 ± 5.8	25.8 ± 3.6	25.7 ± 3.8
Body Mass Index (mean ± SD, kg/m^2^)	22.1 ± 1.9	22.1 ± 1.9	22.4 ± 1.7	22.4 ± 1.7	21.9 ± 1.9	21.7 ± 1.8

### Endpoints

Primary efficacy variable was vaccine-specific antibody titers, including specifically IgG and IgM antibodies.

Secondary variables were seroconversion rate, serum immunoglobulins (Ig A, IgE, IgG and IgM), lymphocytes subsets (total T, naïve and memory T helper and naïve and memory T cytotoxic lymphocytes, B lymphocytes, Natural Killer (NK) cells) and cytokines production (interleukin (IL)-4, IL-6, IL-13, IL-10, IL-12, interferon (IFN)-γ and tumour necrosis factor (TNF)-α).

### Blood analysis

#### Specific immunity

White blood cell (WBC) counts and differential were determined with automated blood cell counters (ADVIA-2120, Siemens, Madrid). Major lymphocyte subset phenotypes were assessed in ethylenediaminetetraacetic acid (EDTA)-treated whole blood samples. For this purpose, blood aliquots were incubated for 30 min at room temperature in the dark with fluorochrome-conjugated monoclonal antibodies with a quadruple immunostaining procedure (CD3/CD8/CD45/CD4, CD45RA/CD45RO/CD8/CD3, CD45RA/CD45RO/CD4/CD3 and CD3/CD16 + 56/CD45/CD19) in order to identify and quantify the following lymphocyte subsets: total T lymphocytes (CD3+), cytotoxic T lymphocytes (CD3 + CD8+), helper T lymphocytes (CD3 + CD4+), B lymphocytes (CD19+), Natural Killer (NK) cells (CD3-CD16 + CD56+), naïve cytotoxic T lymphocytes (CD8 + CD45RA+), memory cytotoxic T lymphocytes (CD3 + CD8 + CD45RO+), naïve helper T lymphocytes (CD4 + CD45RA+), and memory helper T lymphocytes (CD3 + CD4 + CD45RO+) (BD Biosciences, San José, CA, USA). After lysing red blood cells, lymphocytes were analyzed by flow cytometry on a FACScalibur system (BD Biosciences, San José, CA, USA). The lympho-gate was defined on the forward and side scatter patterns of lymphocytes. The analysis protocol gated on lymphocytes stained with PerCP (Peridinin chlorophyll) and/or APC (Allophycocyanin) and the selected population was then analysed with the two remaining colours FITC (Fluorescein isothiocyanate) and PE (Phycoerythrin) to obtain percentages of cell expressing the specific antigens. For memory and naïve subsets, the anchor marker used was annotated in the first place. The results were expressed as the percentage and cell number of mononuclear cells positively stained.

Serum immunoglobulins (Ig) A, IgE, IgG and IgM levels were measured in EDTA-treated whole blood samples by immunoturbidometry.

Specific HAV antibodies were assessed with a competitive Enzyme-Linked ImmunoSorbent Assay (ELISA) kit (DIA.PRO, Italy), both before (V1) and after (V3) the intervention [23]. A cut-off value (negative control + positive control/3) was used to confirm negative or positive Hepatitis A results. The kit detects total anti-HAV IgM and IgG levels (mUI/ml). Seroconversion was defined as the proportion of subjects that change from a negative to a positive result after vaccination, after exclusion of those with positive results before the challenge.

#### Cytokine analysis

Blood was collected in Vacutainer tubes (BD Biosciences) and allowed to clot. Within an hour, plasma was separated by centrifugation at 3500 rpm for 15 min and aliquots were stored at −80 °C. At the end of the study, multiplex magnetic bead array (Merck-Millipore) was performed for the quantification of immune and inflammation-related cytokines: interleukin (IL)-4, IL-6, IL-13, IL-10, IL-12, interferon (IFN)-γ and tumour necrosis factor (TNF)-α. In the case of IL-4 and IL-13, there were 18.03 and 62.5% of undetectable data respectively, which were not included into the statistical analysis.

#### Statistical analysis

Kolmogorow-Smirnov test was performed to evaluate the normality of the variables. For the variables fitting Gaussian distribution, data were expressed as mean and Standard Deviation (SD), and for the non-Gaussian variables data were expressed as median and Interquartil Range (IQR, percentile 25, percentile 75). Logarithmic transformation was used for the following variables not fitting a normal distribution: CD19+ and CD16 + CD56+ lymphocyte subset percentages and CD19+, CD8 + CD45RA+, CD4 + CD45RA+, CD3 + CD8 + CD45RO+, CD3 + CD4 + CD45RO+, and CD16 + CD56+ counts. One-way ANOVA with Bonferroni post-hoc test were performed for normally-distributed variables to evaluate the “group effect” within each visit, and a lineal mixed model of repeated measures was performed to analyse the “visit effect” in the different groups (fixed factor “visit” and random factor “sex”). For those variables not fitting normal distribution (all cytokine variables and IgE levels), non-parametric Kruskal-Wallis test and Mann-Whitney U test were performed for group comparisons within visits and Friedman’s test for paired samples was used for between visit comparisons within the same group. The Chi square test was used to assess seroconversion rates. Data analysis was performed using SPSS v.19 Software. *P* values <0.05 were considered significant.

## Results

Regarding white blood cells counts and differential, no differences were found between groups in each visit, nor within each group along the intervention (Additional file 1: Table S1).

### Effects on specific immunity

Inter-group comparisons showed no significant effect of treatment on lymphocyte subset percentages (Additional file 1: Table S2), but a significant increase in memory T helper lymphocyte counts (CD3 + CD4 + CD45RO+) was found in PRO2 at the end of the intervention (V3) compared to basal values (V1) (*P* = 0.032) (Table 2).

**Table 2:** Lymphocytes subsets (cells/μL) at the beginning (V1), after 2 (V2) and 6 weeks (V3) of intervention

	V1		V2		V3		
	Mean	SD	Mean	SD	Mean	SD	*P* ^*#*^
CD3+ Lymphocytes							
Control	1851	589	1817	626	1865	507	NS
PRO2	1660	522	1695	496	1861	558	NS
PRO1	1812	494	1802	422	1876	401	NS
CD8+ Lymphocytes							
Control	611	229	608	250	605	191	NS
PRO2	512	217	519	236	576	239	NS
PRO1	543	193	536	194	553	171	NS
CD4+ Lymphocytes							
Control	1073	382	1034	420	1104	387	NS
PRO2	992	345	1007	315	1132	395	NS
PRO1	1111	323	1099	280	1160	278	NS
CD19+ Lymphocytes							
Control	248	102	248	98	250	123	NS
PRO2	234	101	251	89	261	105	NS
PRO1	273	104	289	120	285	114	NS
CD3-CD16 + CD56+ Cells							
Control	299	154	272	164	278	169	NS
PRO2	275	138	281	144	236	105	NS
PRO1	328	196	311	172	305	165	NS
CD8 + CD45RA+ Lymphocytes							
Control	336	158	326	170	337	138	NS
PRO2	282	158	290	137	327	159	NS
PRO1	304	136	290	132	309	309	NS
CD3 + CD8 + CD45RO+ Lymphocytes							
Control	291	147	269	113	274	109	NS
PRO2	232	108	235	126	211	133	NS
PRO1	263	110	251	115	256	104	NS
CD4 + CD45RA+ Lymphocytes							
Control	443	245	413	242	451	246	NS
PRO2	405	218	405	204	454	247	NS
PRO1	483	224	452	174	513	205	NS
CD3 + CD4 + CD45RO+ Lymphocytes							
Control	581	195	562	193	630	217	NS
PRO2	**551** ^**a**^	**213**	**594** ^**ab**^	**215**	**660** ^**b**^	**223**	**0.032**
PRO1	565	221	583	196	616	200	NS

Data are expressed as mean ± SD. ^#^Differences among visits within each group, also highlighted in bold. Repeated measures ANOVA with “visit” as fixed factor and “sex” as randomized factor (*P* < 0.05). Different superscripts mean significant differences between visits; Bonferroni test (*P* < 0.05)

Although there were no changes regarding plasma immunoglobulin levels (Additional file 1: Table S3), PRO1 showed significantly higher values of specific HAV antibodies compared to the control group after 6 weeks of intervention (*P* = 0.017) (Table 3). PRO1 HAV-antibody levels were 39% higher compared to the control group titres, while those of PRO2 were only 14.8% higher. Seven volunteers (5.7%) showed positive HAV Ab levels at V1, probably due to an ignored previous contact with the virus, and were thus excluded from the analysis. In addition, there were 5 volunteers with negative titers against HAV specific antibodies after four weeks of vaccination (4.3%); more days might be necessary for these volunteers to produce enough antibodies for a positive response. Therefore, seroconversion rates were 97.3, 92.3 and 97.4% in C, PRO1 and PRO2 respectively, values that were not significantly different.

**Table 3:** Specific HAV antibodies (mIU/mL) at the beginning (V1) and after 6 weeks (V3) of intervention

	Control (*n* = 38)	PRO2 (*n* = 38)	PRO1 (*n* = 37)	*P* ^*#*^
V1	Neg	Neg	Neg	–
V3	**36.23 ± 16.45** ^**a**^	**41.61 ± 15.74** ^**ab**^	**50.54 ± 29.57** ^**b**^	**0.017**

Data are expressed as mean ± SD. ^#^Differences among groups by one-way ANOVA (*P* < 0.05), also highlighted in bold. Different superscripts mean significant differences between visits; Bonferroni test (*P* < 0.05)

### Effects on cytokine levels

No significant differences were found among the different groups at any visit. In addition, serum cytokine levels did not change along the study in the treated groups. However, C group showed an increase in TNF-α values from V1 to V2 (*P* = 0.052) and reaching statistical significance after 6 weeks of intervention (*P* = 0.011 V1 *vs*. V3). Similarly, IL-10 values showed a marginal increase from V1 to V2 (*P* = 0.058), reaching statistical significance compared with V3 (*P* = 0.016; V1 *vs*. V3). However, IFN-γ values decreased during the first two weeks of the intervention V1 to V2 (*P* = 0.037) in this group (Table 4).

**Table 4:** Cytokines (pg/mL) at the beginning (V1), after 2 (V2) and 6 weeks (V3) of intervention

	V1		V2		V3		
	Median	IQR	Median	IQR	Median	IQR	*P* ^*#*^
TNF-α							
Control	**3.43** ^**a**^	**2.56–4.97**	**3.83** ^**ab**^	**2.98–4.76**	**4.03** ^**b**^	**2.80–4.98**	**0.019**
PRO2	4.20	3.38–5.41	4.63	3.66–5.79	4.07	3.22–6.28	NS
PRO1	4.23	3.77–5.43	4.35	3.34–5.79	4.60	3.08–6.28	NS
IFN-γ							
Control	**6.26** ^**a**^	**2.96–10.75**	**6.11** ^**b**^	**3.24–12.02**	**5.97** ^**ab**^	**3.31–12.75**	**0.049**
PRO2	9.47	2.91–17.46	9.28	4.68–15.94	8.51	5.05–13.77	NS
PRO1	7.55	3.73–16.36	7.75	4.10–21.40	9.15	4.95–15.72	NS
IL-4							
Control	6.65	3.03–25.23	9.04	2.04–24.57	9.12	2.34–22.38	NS
PRO2	2.95	0.21–14.60	4.93	0.61–22.78	3.75	1.46–22.42	NS
PRO1	6.96	1.08–21.74	6.11	1.29–29.09	10.22	2.16–15.38	NS
IL-13							
Control	3.47	0.39–8.46	2.57	0.52–9.57	2.73	1.28–6.18	NS
PRO2	2.73	0.19–5.44	2.50	0.70–8.77	3.24	0.33–8.55	NS
PRO1	2.79	0.93–8.50	2.33	0.96–7.21	1.22	0.10–4.73	NS
IL12p70							
Control	3.66	1.22–5.07	4.35	1.85–7.04	4.41	2.41–7.91	NS
PRO2	5.09	1.40–10.07	5.33	2.72–8.17	4.46	3.03–9.37	NS
PRO1	5.00	2.67–9.88	4.96	2.17–13.51	5.41	2.79–12.86	NS
IL-10							
Control	**21.85** ^**a**^	**11.87–32.52**	**24.77** ^**ab**^	**16.26–35.42**	**25.23** ^**b**^	**16.74–42.47**	**0.030**
PRO2	27.07	13.44–44.61	29.29	19.16–53.47	30.37	19.37–40.75	NS
PRO1	31.53	20.13–48.29	33.90	16.39–49.96	34.23	16.97–57.00	NS
IL-6							
Control	1.10	0.46–1.81	1.15	0.57–2.70	1.17	0.56–2.10	NS
PRO2	2.11	0.76–3.30	2.66	0.63–3.72	1.87	0.68–3.86	NS
PRO1	1.70	0.79–3.46	1.31	0.79–3.46	1.82	0.97–2.97	NS

Data are expressed as median and interquartile range (IQR. percentile 25-percentil 75). # Differences among visits within each group by Friedman’s test for related samples, also highlighted in bold. Different superscripts mean significant differences between visits (Friedman’s test; *P* < 0.05)

## Discussion

Specific strains of probiotics interact with host cells and intestinal microbiota, and could thus have a role as immune modulators not only in patients with disease but also in healthy subjects under specific circumstances, such as an immune challenge as performed in this study. In fact, our findings showed that the consumption of Lc during two weeks before vaccination seems to be associated with an enhanced antibody response. However, the regular intake of this strain prior and following the challenge did not increase antibody titres but led to an increase in memory T helper lymphocytes.

*L. coryniformis* CECT5711 intake did not change the percentage and number of total T lymphocytes, including helper and cytotoxic T cells, B lymphocytes and NK cells. On the contrary, *L. coryniformis* CECT5711 in combination with *L. gasseri* CECT5714 consumed in a dose of 10^6^ cfu/g each during three months, led to an enhancement of NK cells in allergic children [24]. The effect of this combination on NK cells was also observed in healthy subjects after two weeks of treatment [25]. In agreement with the lack of effect on lymphocyte subsets in our results, several studies with different *Lactobacillus* strains supplementation in healthy individuals have also shown no significant effects on CD3+, CD4+, CD8+ and CD19+ percentages [25, 26]. Therefore, probiotics may affect the activity of certain immune cell types and not others, being the differences due to probiotic strain specificity.

There was an increase in memory T helper lymphocytes (CD3 + CD4 + CD45RO+) after the vaccine challenge in the group that consumed the probiotic strain during 6 weeks (PRO2), which might be linked to the establishment of immunological memory against the viral antigen. However, further research should be aimed to confirm the production of HAV specific memory CD4+ T cell clones, since specific memory cells can be reactivated after a secondary microbial exposure and are related to a long term protection [27]. The effect on lymphocyte subsets of a vaccination protocol against influenza virus while consuming *L. fermentum* CECT5716 (10^10^ cfu/d) was found to increase the percentage of helper and cytotoxic T cells after a two week challenge both in the placebo and treated groups [15]. The different timing in lymphocyte subset analysis between studies might explain why we did not observe the same increase in T cells, since we measured it after four weeks of vaccination. In addition, the nature of the vaccine antigen (bacterial or protein/ live or non live vaccines) and its administration could be main determinants in the immune response elicited after a vaccine shot [8] and thus relevant to evaluate differences between studies.

The specific production of antibodies in response to vaccination is considered as a useful measure which directly correlates with specific protection and the ‘gold-standard’ to determine the influence of probiotics on immunity [28]. In this regard, studies in animals and humans have shown the potential of probiotics to act as immune adjuvants [9], with an effect on specific vaccine antibody production in susceptible population groups such as children [29–32] and elderly people [33, 34].

The current study is the first to use a vaccine challenge to assess the immune modulation exerted by the *L. coryniformis* CECT5711 strain in healthy adults. The effect of probiotics as vaccine adjuvants has previously been shown with other strains. The oral administration of the *L. fermentum* CECT5716 strain has been found to enhance the immune response of an anti-influenza vaccine and provide systemic protection from infection by increasing antigen specific IgA, but not IgG levels in 50 subjects [15]. In addition, the intake of *B. animalis* ssp*. lactis* and *L. paracasei* ssp*. paracasei* for 6 weeks have been shown to increase influenza vaccine-specific serum IgG measured 4 weeks after vaccination compared to placebo in 211 adults [10]. However, no effects in influenza A–specific IgG1 and IgG3 seroconversion measured three weeks after the vaccine were observed after the consumption of *L. paracasei subsp. Paracasei* 431 in 1066 healthy subjects. Protection rate after a seasonal influenza vaccine varies from year to year and these studies were performed in different campaigns, so the differences in the viral challenges between campaigns could also contribute to the different results found among these studies. Since rates up to 99% seroprotection were observed in Jespersen’s et al. study, the authors hypothesized that it might be difficult to observe a further increase in protection rates due to the probiotic intake [35]. In the current study, the consumption of the probiotic strain during 2 weeks in PRO1 induced an increase in Hepatitis A-specific antibodies after the vaccine, compared to the control group (*P* = 0.017). The immunological mechanism elicited after a vaccine challenge involves the activation of immature dendritic cells (DCs) by local inflammation, which take up the vaccine antigens and migrate to draining lymph nodes where the activation of T and B lymphocytes will take place. T cell help induces B cell differentiation into Ig secreting plasma cells that produce low-affinity IgG antibodies during this primary antibody response. Therefore, *L. coryniformis* CECT5711 might act as a coadjuvant of the antibody response in a vaccination protocol in healthy subjects when consumed before the vaccine challenge. This improvement in vaccine response could be relevant, since there is a low percentage of supposedly healthy individuals who exhibit an impaired response to the immune challenge of this vaccine and sometimes it needs an extra booster. In addition, EFSA states that “the stimulation of protective antibody titters in response to vaccination could be used to substantiate a health claim on the function of the immune system related to defence against pathogens” [11].

The fact that the increase of specific antibodies was not significant in PRO2 compared to placebo is difficult to explain. It might suggest that the probiotic consumption two weeks before vaccination works better as adjuvant of the humoral response than the continuation of *L. coryniformis* administration after vaccination; however, the level of specific HAV antibodies in PRO2 was at an intermediate level between the other two groups. In this sense, the continuous intake of this strain during 6 weeks in PRO2 could induce a higher T cell expansion, which is the main determinant of memory T cell responses and a weaker antibody response compared to the response induced when the strain was consumed only during 2 weeks in PRO1. In addition, regulatory T cell responses should be further evaluated in another study since an inverse relationship was observed between Tregs and antibody responses [36]. In fact, an enhancement of anti-cancer vaccine responses was observed in healthy adults following Tregs depletion [37]. No effects in inflammatory cytokines were seen when consuming the probiotic strain, in contrast to the placebo group, which showed higher levels of the pro-inflammatory TNF-α cytokine and the anti-inflammatory IL-10 four weeks after the vaccine, probably as an on-going reaction to the challenge [38]. In this context, the probiotic intake might have modulated the cytokine response to the vaccine. Although the early cytokine response after the shot was not evaluated, we could speculate that the probiotic intake might favour an early recovery of immunological homeostasis. On this basis, since mixed results were found depending on the timing and length of supplementation with the probiotic in relation to the viral challenge, we consider that one limitation of this study was the lack of certain additional times and immune measurements, such as innate immunity 2 weeks after vaccination and regulatory T cells at 4 weeks post-vaccination, in order to ascertain the role of the lactobacillus strain as an adjuvant for HAV vaccine.

## Conclusions

*L. coryniformis* CECT5711 strain consumed two weeks before the vaccine led to an increase of total HAV antibody titres compared to placebo. This supports the hypothesis that the consumption of this strain might have a clinical benefit in protection from future infections. However, an independent study is warranted to clarify the adjuvant effects obtained with specific protocols of Lc supplementation and the mechanisms involved.

## Additional file

Additional file 1: Table S1. White blood cells (cells/μL) expressed as mean ± SD at the beginning (V1), after 2 weeks (V2) and after 6 weeks (V3) of intervention in all groups. **Table S2** Lymphocyte subsets (%) in blood samples expressed as mean ± SD at the beginning (V1), after 2 weeks (V2) and after 6 weeks (V3) of intervention in all groups. **Table S3** Immunoglobulin levels in plasma samples expressed as mean ± SD at the beginning (V1), after 2 weeks (V2) and after 6 weeks (V3) of the intervention in all groups. Description of data: Data from analyses which did not show statistical significance. (DOCX 36 kb)

## Abbreviations

APC: Allophycocyanin
BMI: Body Mass Index
CFDA-SE: Carboxyfluorescein diacetate N-succinimidyl ester
cfu: Colony forming units
DCs: Dendritic cells
DMSO: Dimethyl sulfoxide
EDTA: Ethylenediaminetetraacetic acid
EFSA: European Food Safety Agency
ELISA: Enzyme-Linked ImmunoSorbent Assay
FITC: Fluorescein isothiocyanate
HAV: Hepatitis A virus
IFN: Interferon
Ig: Immunoglobulins
IL: Interleukin
IQR: Interquartil Range
Lc: *Lactobacillus coryniformis*
NK: Natural killer
PBMCs: Peripheral blood mononuclear cells
PE: Phycoerythrin
PerCP: Peridinin chlorophyll
QPS: Qualified Presumption of Safety
RPMI: Roswell Park Memorial Institute
SD: Standard desviation
TGF: Transforming growth factor
TNF: Tumour necrosis factor
V: Visit
WBC: White blood cells
WHO: World Health Organization
wk: Week
