# Erratum to: Evaluation of *Lactobacillus coryniformis* CECT5711 strain as a coadjuvant in a vaccination process: a randomised clinical trial in healthy adults

**DOI:** 10.1186/s12986-017-0192-4

**Published:** 2017-06-14

**Authors:** Noemí Redondo, Esther Nova, Alina Gheorghe, Ligia Esperanza Díaz, Aurora Hernández, Ascensión Marcos

**Affiliations:** Immunonutrition Group (Metabolism and Nutrition Department) – Instituteof Food Science, Technology and Nutrition, Spanish National ResearchCouncil (ICTAN-CSIC), José Antonio Novais St. 10, 28040 Madrid, Spain

## Erratum

Following publication of the original article [1], it was agreed amongst the authors that Dr. Jorge R. Mujico should be acknowledged as an author, having contributed to the design, data acquisition and analysis in the article. Having left the research group in January 2014, with no further contact or participation in the drafting or approval of the manuscript, Dr. Mujico was not included in the authorship given in the published paper.
