# Supplementary material for: Evaluation of Lactobacillus coryniformis CECT5711 strain as a coadjuvant in a vaccination process: a randomised clinical trial in healthy adults
Source: Nutr Metab (Lond). 2017 Jan 5;14:2. doi: 10.1186/s12986-016-0154-2 (PMC5217323; doi:10.1186/s12986-016-0154-2)
Supplement: Additional file 1: Table S1. — White blood cells (cells/μL) expressed as mean ± SD at the beginning (V1), after 2 weeks (V2) and after 6 weeks (V3) of intervention in all groups. Table S2 Lymphocyte subsets (%) in blood samples expressed as mean ± SD at the beginning (V1), after 2 weeks (V2) and after 6 weeks (V3) of intervention in all groups. Table S3 Immunoglobulin levels in plasma samples expressed as mean ± SD at the beginning (V1), after 2 weeks (V2) and after 6 weeks (V3) of the intervention in all groups. Description of data: Data from analyses which did not show statistical significance. (DOCX 36 kb) [file 12986_2016_154_MOESM1_ESM.docx]

**Additional files**

Table S1. White blood cells (cells/µL) expressed as mean ± SD at the beginning (V1), after 2 weeks (V2) and after 6 weeks (V3) of intervention in all groups.

|  | **V1** | | **V2** | | **V3** | |  |
| --- | --- | --- | --- | --- | --- | --- | --- |
|  | **Mean** | **SD** | **Mean** | **SD** | **Mean** | **SD** | ***P#*** |
|  |  |  |  |  |  |  |  |
| **Leucoytes (cells/µL)** |  |  |  |  |  |  |  |
| Control | 6548 | 1329 | 6806 | 1780 | 6609 | 1849 | NS |
| PRO2 | 6521 | 1417 | 6450 | 1266 | 6863 | 1888 | NS |
| PRO1 | 6388 | 1346 | 6305 | 1085 | 6389 | 1336 | NS |
| **Lymphocytes (%)** | | | | | | | |
| Control | 37.82 | 8.56 | 36.82 | 11.17 | 38.77 | 9.85 | NS |
| PRO2 | 35.26 | 9.22 | 35.94 | 7.79 | 36.34 | 7.93 | NS |
| PRO1 | 39.27 | 7.63 | 38.76 | 6.28 | 39.48 | 6.98 | NS |
| **Lymphocytes (cells/µL)** |  |  |  |  |  |  |  |
| Control | 2470 | 702 | 2395 | 713 | 2455 | 619 | NS |
| PRO2 | 2283 | 610 | 2255 | 542 | 2446 | 663 | NS |
| PRO1 | 2441 | 591 | 2419 | 545 | 2501 | 537 | NS |
| **Monocytes (%)** | | | | | | | |
| Control | 6.37 | 1.40 | 6.16 | 1.58 | 6.63 | 1.76 | NS |
| PRO2 | 6.38 | 1.44 | 6.21 | 1.30 | 6.11 | 1.18 | NS |
| PRO1 | 6.29 | 1.40 | 6.37 | 1.27 | 6.71 | 1.28 | NS |
| **Monocytes (cells/µL)** | | | | | | | |
| Control | 420 | 1212 | 419 | 148 | 439 | 176 | NS |
| PRO2 | 408 | 115 | 396 | 121 | 420 | 143 | NS |
| PRO1 | 392 | 91.23 | 402 | 96.01 | 427 | 111 | NS |
| **Neutrophils (%)** | | | | | | | |
| Control | 52.61 | 8.87 | 53.77 | 11.50 | 51.45 | 9.85 | NS |
| PRO2 | 54.13 | 8.8 | 51.76 | 7.57 | 53.99 | 8.1 | NS |
| PRO1 | 50.99 | 7.56 | 51.43 | 6.39 | 50.19 | 7.28 | NS |
| **Neutrophils (cells/µL)** | | | | | | | |
| Control | 3502 | 999 | 3796 | 1712 | 3512 | 1682 | NS |
| PRO2 | 3564 | 1121 | 3472 | 940 | 3770 | 1572 | NS |
| PRO1 | 3279 | 959 | 3273 | 721 | 3265 | 951 | NS |
| **Eosinophils (%)** | | | | | | | |
| Control | 2.63 | 1.70 | 2.81 | 1.70 | 2.59 | 1.53 | NS |
| PRO2 | 2.80 | 1.92 | 2.81 | 1.99 | 2.70 | 2.22 | NS |
| PRO1 | 3.08 | 1.61 | 3.13 | 1.48 | 3.05 | 1.55 | NS |
| **Eosinophils (cells/µL)** | | | | | | | |
| Control | 168 | 106 | 194 | 147 | 167 | 101 | NS |
| PRO2 | 184 | 149 | 182 | 148 | 190 | 183 | NS |
| PRO1 | 199 | 125 | 200 | 109 | 201 | 123 | NS |
| **Basophils (%)** | | | | | | | |
| Control | 0.64 | 0.24 | 0.63 | 0.26 | 0.58 | 0.24 | NS |
| PRO2 | 0.58 | 0.24 | 0.69 | 0.29 | 0.56 | 0.22 | NS |
| PRO1 | 0.64 | 0.25 | 0.67 | 0.23 | 0.59 | 0.22 | NS |
| **Basophils (cells /µL)** | | | | | | | |
| Control | 41.26 | 15.06 | 42.05 | 16.80 | 37.35 | 15.34 | NS |
| PRO2 | 37.49 | 16.63 | 43.73 | 19.41 | 37.81 | 16.39 | NS |
| PRO1 | 40.65 | 17.51 | 42.11 | 15.53 | 37.21 | 14.90 | NS |

**#General Mixed Model or Friedman’s test were used to assess differences between visits within the same group for normal and non-normally distributed variables, respectively.**

Table S2. Lymphocyte subsets (%) in blood samples expressed as mean ± SD at the beginning (V1), after 2 weeks (V2) and after 6 weeks (V3) of intervention in all groups.

|  | **V1** | | **V2** | | **V3** | |  |
| --- | --- | --- | --- | --- | --- | --- | --- |
|  | **Mean** | **SD** | **Mean** | **SD** | **Mean** | **SD** | ***P*** |
| **CD3+ Lymphocytes** | | | | | | | |
| Control | 75.22 | 6.71 | 75.03 | 7.72 | 75.86 | 7.17 | NS |
| PRO2 | 73.45 | 7.41 | 73.69 | 6.73 | 76.20 | 5.59 | NS |
| PRO1 | 73.20 | 7.58 | 72.95 | 6.25 | 74.46 | 6.61 | NS |
| **CD8+ Lymphocytes** | | | | | | | |
| Control | 24.97 | 6.08 | 25.06 | 6.42 | 24.8 | 5.92 | NS |
| PRO2 | 22.57 | 6.68 | 22.34 | 6.83 | 23.47 | 6.58 | NS |
| PRO1 | 21.90 | 5.25 | 21.51 | 5.17 | 21.80 | 4.70 | NS |
| **CD4+ Lymphocytes** | | | | | | | |
| Control | 43.39 | 6.39 | 42.38 | 7.67 | 44.53 | 7.36 | NS |
| PRO2 | 43.77 | 7.26 | 43.91 | 7.37 | 46.23 | 7.00 | NS |
| PRO1 | 44.85 | 6.45 | 44.48 | 6.07 | 46.12 | 6.82 | NS |
| **CD19+ Lymphocytes** | | | | | | | |
| Control | 10.34 | 3.70 | 10.72 | 3.97 | 10.16 | 3.72 | NS |
| PRO2 | 10.39 | 3.13 | 11.03 | 2.96 | 10.73 | 3.14 | NS |
| PRO1 | 10.99 | 3.50 | 11.64 | 3.88 | 11.26 | 3.73 | NS |
| **CD3- CD16+CD56+ NK cells** | | | | | | | |
| Control | 12.13 | 5.48 | 11.61 | 6.45 | 11.41 | 6.26 | NS |
| PRO2 | 12.88 | 6.62 | 12.48 | 6.21 | 10.20 | 5.09 | NS |
| PRO1 | 13.13 | 6.49 | 12.42 | 5.51 | 11.79 | 5.34 | NS |
| **CD8+CD45RA+ Lymphocytes** | | | | | | | |
| Control | 54.78 | 11.29 | 52.60 | 11.58 | 55.36 | 12.55 | NS |
| PRO2 | 54.83 | 11.30 | 55.56 | 9.87 | 56.24 | 11.62 | NS |
| PRO1 | 55.53 | 13.45 | 54.08 | 12.53 | 55.48 | 12.52 | NS |
| **CD3+CD8+CD45RO+ Lymphocytes** | | | | | | | |
| Control | 12.55 | 6.93 | 15.32 | 5.33 | 14.95 | 5.20 | NS |
| PRO2 | 14.05 | 5.20 | 13.87 | 4.66 | 13.72 | 5.17 | NS |
| PRO1 | 14.86 | 5.53 | 14.11 | 6.02 | 13.92 | 5.48 | NS |
| **CD4+CD45RA+ Lymphocytes** | | | | | | | |
| Control | 39.78 | 10.65 | 38.16 | 9.40 | 39.54 | 11.58 | NS |
| PRO2 | 39.22 | 12.73 | 39.36 | 10.25 | 38.97 | 10.81 | NS |
| PRO1 | 42.81 | 12.88 | 41.09 | 12.17 | 43.56 | 12.99 | NS |
| **CD3+CD+CD45RO+ Lymphocytes** | | | | | | | |
| Control | 31.98 | 6.95 | 31.7 | 6.31 | 34.22 | 8.23 | NS |
| PRO2 | 33.36 | 8.28 | 34.97 | 6.94 | 35.75 | 7.51 | NS |
| PRO1 | 31.61 | 9.98 | 32.32 | 7.46 | 33.07 | 8.96 | NS |
| **#General Mixed Model or Friedman’s test were used to assess differences between visits within the same group for normal and non-normally distributed variables, respectively.** | | | | | | | |

Table S3. Immunoglobulin levels in plasma samples expressed as mean ± SD at the beginning (V1), after 2 weeks (V2) and after 6 weeks (V3) of the intervention in all groups.

|  | **V1** | | **V2** | | **V3** | |  |
| --- | --- | --- | --- | --- | --- | --- | --- |
|  | **mean** | **SD** | **mean** | **SD** | **mean** | **SD** | ***P*** |
| **IgG (mg/dl)** | | | | | | | |
| Control | 1096 | 171 | 1070 | 162 | 1075 | 176 | NS |
| PRO2 | 1109 | 206 | 1099 | 203 | 1088 | 179 | NS |
| PRO1 | 1084 | 224 | 1049 | 206 | 1069 | 207 | NS |
| **IgA (mg/dL)** | | | | | | | |
| Control | 221 | 84.54 | 212 | 79.89 | 214 | 79.02 | NS |
| PRO2 | 202 | 76.98 | 199 | 74.73 | 202 | 72.54 | NS |
| PRO1 | 215 | 99.97 | 203 | 85.63 | 203 | 85.86 | NS |
| **IgM (mg/dL)** | | | | | | | |
| Control | 157 | 84.06 | 148 | 69.67 | 153 | 79.66 | NS |
| PRO2 | 148 | 53.13 | 140 | 47.01 | 144 | 53.88 | NS |
| PRO1 | 160 | 84.21 | 159 | 88.25 | 165 | 89.42 | NS |
| **Median IQR Median IQR Median IQR** | | | | | | | |
| **IgE total (UI/mL)** | | | | | | | |
| Control | 16 | (8-77) | 17 | (6-77) | 17 | (7-76.5) | NS |
| PRO2 | 34 | (11-100) | 31 | (10.5-115) | 35 | (12.5-105) | NS |
| PRO1 | 34.5 | (11-98.5) | 33.5 | (10.5-95.5) | 37 | (9-72.75) | NS |

Interquartile range (IQR)

**#General Mixed Model or Friedman’s test were used to assess differences between visits within the same group for normal and non-normally distributed variables, respectively.**
